# Comparable Long-Term Efficacy of Lopinavir/Ritonavir and Similar Drug-Resistance Profiles in Different HIV-1 Subtypes

**DOI:** 10.1371/journal.pone.0086239

**Published:** 2014-01-27

**Authors:** Zehava Grossman, Jonathan M. Schapiro, Itzchak Levy, Daniel Elbirt, Michal Chowers, Klaris Riesenberg, Karen Olstein-Pops, Eduardo Shahar, Valery Istomin, Ilan Asher, Bat-Sheva Gottessman, Yonat Shemer, Hila Elinav, Gamal Hassoun, Shira Rosenberg, Diana Averbuch, Keren Machleb-Guri, Zipi Kra-Oz, Sara Radian-Sade, Hagit Rudich, Daniela Ram, Shlomo Maayan, Nancy Agmon-Levin, Zev Sthoeger

**Affiliations:** 1 Public Health School, Tel-Aviv University, Tel-Aviv, Israel; 2 Sheba Medical Center, Ramat Gan, Israel; 3 Kaplan Medical Center, Rehovot, Israel; 4 Meir Medical Center, Kfar Saba, Israel; 5 Soroka Medical Center, Beer Sheva, Israel; 6 Hadassah Medical Center, Jerusalem, Israel; 7 Rambam Medical Center, Haifa, Israel; 8 Hillel Yaffe Medical Center, Hadera, Israel; 9 National HIV Reference Lab, PHL, MOH, Ramat Gan, Israel; University of Pittsburgh, United States of America

## Abstract

**Background:**

Analysis of potentially different impact of Lopinavir/Ritonavir (LPV/r) on non-B subtypes is confounded by dissimilarities in the conditions existing in different countries. We retrospectively compared its impact on populations infected with subtypes B and C in Israel, where patients infected with different subtypes receive the same treatment.

**Methods:**

Clinical and demographic data were reported by physicians. Resistance was tested after treatment failure. Statistical analyses were conducted using SPSS.

**Results:**

607 LPV/r treated patients (365 male) were included. 139 had HIV subtype B, 391 C, and 77 other subtypes. At study end 429 (71%) were receiving LPV/r. No significant differences in PI treatment history and in median viral-load (VL) at treatment initiation and termination existed between subtypes. MSM discontinued LPV/r more often than others even when the virologic outcome was good (p = 0.001). VL was below detection level in 81% of patients for whom LPV/r was first PI and in 67% when it was second (P = 0.001). Median VL decrease from baseline was 1.9±0.1 logs and was not significantly associated with subtype. Median CD4 increase was: 162 and 92cells/µl, respectively, for patients receiving LPV/r as first and second PI (P = 0.001), and 175 and 98, respectively, for subtypes B and C (P<0.001). Only 52 (22%) of 237 patients genotyped while under LPV/r were fully resistant to the drug; 12(5%) were partially resistant. In48%, population sequencing did not reveal resistance to any drug notwithstanding the virologic failure. No difference was found in the rates of resistance development between B and C (p = 0.16).

**Conclusions:**

Treatment with LPV/r appeared efficient and tolerable in both subtypes, B and C, but CD4 recovery was significantly better in virologically suppressed subtype-B patients. In both subtypes, LPV/r was more beneficial when given as first PI. Mostly, reasons other than resistance development caused discontinuation of treatment.

## Introduction

Subtype B is the predominant HIV-1 subtype in the resource rich countries, but most of those infected worldwide carry non-B virus [1], [2]. HIV subtypes show characteristic patterns of amino acids expressed at specific positions throughout the viral genome [3]–[7]. Differences between subtypes in the protease and reverse transcriptase genes have the potential to alter responses to combination antiretroviral treatment (cART). Mutations at 12 amino-acid positions in the protease can be designated primary or major resistance mutations (D30N, V32I, M46I/L, I47A, G48V, I50V/L, I54L/M/V, L76V, V82A/F/S/T, I84A/V, N88S, and L90M [8]–[10]. Several other mutations are considered secondary or minor, although definitions can vary. Although no major mutations occur as polymorphisms in wild-type subtype-C HIV-1, several secondary mutations associated with drug failure are found at high frequency in viruses from drug-naive subtype-C patients (*e.g.*, M36I and I93L) [11], [12]. These differences in baseline sequence between subtypes may result in the evolution of drug resistance along distinct mutational pathways, or in the incidence of different pathways [13]–[18]. Thus subtle genetic differences may have clinical relevance when considering long-term treatment strategies for patients infected with different subtypes.

Lopinavir co-formulated with ritonavir, (LPV/r, Kaletra®) is a widely used protease inhibitor (PI). WHO latest guidelines [19] recommended LPV/r-based regimen as first-line cART for all HIV-infected children under the age of three in developing countries and as the PI of choice in second-line treatment [19]–[21]. In Africa, a particular advantage of this drug over NNRTIs is that some Africans have slower clearance of NNRTIs than Caucasians rendering them more susceptible to resistance development during treatment interruptions [22]–[25] and it may also reduce the incidence of malaria among children receiving it [26]. Several studies reported treatment outcome in developing countries (*e.g.*, [27]–[32]) typically with only limited genotypic information regarding patients failing LPV/r (*e.g.*, [31], [32]). The first larger-scale study, by van Zyl *et al.*, who provided and analyzed such genotypic data from 490 LPV/r-failing patients in South-Africa, has just appeared [33].

Mutations conferring reduced susceptibility of the virus to LPV/r are well documented (amino acids at positions 10, 16, 20, 24, 32, 33, 34, 36, 43, 46, 47, 48, 50, 53, 54, 58, 71, 73, 74, 76, 82, 84, 89, and 90) [34], [35]. Four genotypic resistance-evaluation algorithms were developed for interpretation of genotypic data for this drug [36]–[39] and used in clinical practice. Both the number of mutations that contribute to resistance and the combinations in which they appear have been studied [40]–[45], but a broad comparison of accumulation of mutations and long-term treatment outcome in the different subtypes is still limited [32], [33], [46], [47]. Analysis of potential differences is confounded by dissimilarities in the conditions existing in different countries. To the best of our knowledge, only one study [48], by Barber *et al.*, compared LPV/r treatment outcome of B and C patients where both groups were treated under equal conditions in a developed country.

In Israel, subtypes B, C and A/AE, are prevalent [49]–[53]. cART is available to all citizens, and genotypic resistance testing is performed to guide treatment decisions [9]. This provides an opportunity to compare the impact of any given drug regimen over the long-term on patients infected with the different subtypes while treated under relatively similar conditions, unlike differences in the conditions of treatment which often exist between B and non-B infected patients. Very few patients were treated with LPV/r in Israel before 2001, when the drug was approved by Health authorities and became part of standard care regimens. Since atazanavir and darunavir were not available in Israel during the study period, LPV/r was the main PI given to drug naive patients. In this study we retrospectively followed a cohort of 607 LPV/r treated patients. The aim was to evaluate potential differences in the clinical outcome and resistance pathways between patients infected with different subtypes, mainly C *versus* B, following the introduction of LPV/r.

Because of the heterogeneity of the patient population and the retrospective nature of the study, a subtype-based comparison of treatment outcomes, particularly in terms of viral-load suppression and CD4 gains, might be confounded by several other factors that potentially influence the results. The median value of several parameters, including age, follow-up period, and time on LPV/r were similar in subtype B and C, and thus not confounding. The outcome of treatment significantly depended, in addition to subtype, on whether the patients had previous PI treatment experience or not, but here too, the proportion of patients for whom LPV/r was first PI was similar in the three subtype groups.

## Materials and Methods

### Patients

Six of the seven AIDS-treating centers in Israel participated in this retrospective study. All patients (607) who received LPV/r for at least 3 months before August 2007 with documented treatment dates and known HIV RNA viral load (VL) levels and CD4+ T-cell counts (CD4 Counts) at starting and stopping/end dates were included. Patients were stratified according to subtype, gender, PI experience prior to LPV/r, and to whether they were on LPV/r treatment or had stopped it during the study. As subtype B patients were mainly MSM, this group of patients was also compared to subtype-C males. The study was approved by the local Ethics committees of the different institutions.

### Clinical specimens and database

Plasma VL levels and CD4 counts were determined by the local hospital laboratories at the different clinical centers as part of routine follow-up. The VL levels and CD4 counts at the beginning and the end of LPV/r treatment, as well as demographic data and adverse events were reported by the treating physicians using standard forms. HIV drug resistance genotypic testing was performed centrally at the National HIV Reference Laboratory as part of the standard of care for patients failing treatment. Data were stored in an anonymous database. HIV genotypes were determined by comparing the sequences to those in Stanford University HIV Drug Resistance Database (Stanford database) Sierra Webservice (http://hivdb.stanford.edu/pages/webservices, version 6.3.1 (last updated 09/20/13 [54]), directed by Robert Shafer [55]). Subtyping was performed using the Rega Subtyping Tool, version 2.0; revised 10/03/2006 [56].

### HIV-1 RNA extraction, viral load measurement and sequencing

Three commercial HIV-1 viral load assays were used by the different AIDS centers: Cobas Amplicor human immunodeficiency virus type 1 (HIV-1) Monitor test, version 1.5 (Roche Molecular Systems, Inc., Branchburg, NJ); the Cobas AmpliPrep/Cobas TaqMan HIV-1 test (Roche Molecular Systems, Inc.); and real-time nucleic acid sequence-based amplification (NASBA) HIV-1 assay (NucliSensEasyQ; bioMerieux, Boxtel, The Netherlands). The assays were performed according to the manufacturer's instructions. The detection level of the least sensitive test was 400 copies/ml and therefore results below this value were considered lower than detection level (LDL). The genotyping tests were all performed at the National HIV Reference Laboratory (NHRL). Viral RNA was isolated from patient blood plasma using the BioMerieux automatic extractor (Easy MAG) according to manufacturer's instructions. The Protease gene (codons 4–99) and RT gene (codons 38–247) were sequenced using Siemens' True-Gene™ kit as described before [12]. Profiles of resistance to the different drugs were determined according to the Stanford database (http://hivdb.stanford.edu/; version 6.3.1; last updated 09/20/13 [54]).

### Statistical analysis

Chi-square test and Fisher's two-tailed exact test were used for analysis of discrete data (e.g., mutation frequencies) and Bonferroni's correction was applied to multiple comparisons of mutations frequency. T-test and one way Anova test were used in comparing continuous clinical data (e.g., viral load and CD4 counts). A square root transformation was applied to the CD4 counts to approach a normal distribution for this variable and the t-test and Anova test were performed on the transformed values.Logistic regression analysis was implemented to predict viral load below detection level at the end of the study. All statistical analyses were performed using SPSS® (version 21.0). Data are presented as median [range] unless otherwise stated. Results are considered statistically significant when *p*<0.05.

### Ethics statement

The retrospective analysis of clinical and laboratory data, which were obtained from the medical charts of HIV-1 patients attending the different Medical Centers, was approved by the respective ethical committees. Specifically, permission was granted by the Kaplan Ethical Committee to analyze such data without the need of a signed informed consent by the patients. The samples obtained at the Sheba Medical Center that were used in this study belonged to patients who had signed an informed consent agreeing to participate in a range of studies.

### Gb-accession numbers of used sequences:

AY529598, KC184165, KC184166, KC184169, KC184185, KC184186, KC184219, KC184325, KC184345, KC184354, KC184392, KC213492, KC213521, KC213541, KC213556, KC213581, KC21358, KC213589, KC213607, KC213609, KC213612, KC213656, KC213662, KC213664, KC213665, KC213666, KC213683, KC213684 and KF134929 - KF135178.

## Results

### Patients

Six hundred and seven patients (365 males and 242 females) from six AIDS clinics in northern, central and southern Israel were included. A summary of patient characteristics can be found in Table 1. One hundred and thirty nine were infected with HIV subtype B, 391 with subtype C, and 77 with other (non-BC) subtypes. Transmission routes, gender and countries of origin differed for the three subtype groups (*p*<0.001). Other parameters, including median age (38.1±0.5 years), median time from diagnosis to starting LPV/r treatment (5.2±0.2 years), median time on LPV/r (23.7±0.8 months) and the fraction of patients for whom LPV/r was first PI were similar in the B and C groups (*p* = 0.1 to *p* = 1) but the median time from diagnosis to LPV/r treatment initiation for the non-BC group was shorter (2.5±0.5 years; p<0.001) and a larger fraction of these patients received LPV/r as first PI (p = 0.006).

**Table 1:** Patient classification.

	Total	B	C	Non-BC	*p*
Female (%)	242 (40)	20 (14)	199(51)	23 (30)	<0.001
Male (%)	365 (60)	119 (86)	192 (49)	54 (70)	<0.001
Age (years); median ± SEM	38.2±0.5	38.5±0.9	38.1±0.7	37.7±1.3	0.2
MSM (%)	75 (12)	72 (52)	1 (0.3)	2 (3)	<0.001
Hetero (%)	422 (70)	37 (27)	362 (93)	23 (30)	<0.001
IVDU (%)	61 (10)	14 (10)	4 (1)	43 (56)	<0.001
Others (%)	49 (8)	16 (11)	24 (6)	9 (11)	0.06
Principal Birth place (%)	Ethiopia (61)	Israel (78)	Ethiopia (94)	FSU (51)	<0.001
Follow up (years); median ± SEM	5.2±0.2	6.3±0.5	5.6±0.3	2.5±0. 5	0.001– 0.6
Time on LPV/r (months); median ± SEM	23.7±0.8	24.4±1.6	23.1±1.0	27.0±2.1	0.9
Patients on LPV/r as first PI (%)	49	49	46	68	0.006–1

FSU – Former Soviet Union; IVDU – Intravenous drug users; MSM – Men who have sex with men; SEM – standard error of mean.

### Drug-combination treatment, treatment interruption and side effects

Median LPV/r treatment time was 23.7±0.8 months (range 3 to 95). For 305 patients (50.2%) LPV/r was the first administrated PI; 140 (46%) of those were naive to prior cART. The most common backbone regimens were ZDV+3TC (65.4%), 3TC plus either ddI or d4T or ABC (13.4%), and ddI plus d4T (9.8%). At the end of the study 429 patients (71%) were receiving LPV/r (“ongoing” group) and 177 had discontinued; of those, 13 died. There was no statistical difference between the subtype B and C or non-BC populations in number of former PIs (zero or more), and number of past regimens, but for a significantly larger fraction of non-BC patients (74%) LPV/r was first PI (p = 0.006). Among patients who were treated with other PIs about 30% received nelfinavir, 30% received indinavir, 30% received both and 10% were treated with other PIs (mainly saquinavir). The distribution of treatment regimens was similar in all subtypes groups.

The various side effects reported by clinicians are shown in Table 2. Sixty patients (10%) had reported side effects. The predominant side effects reported were gastrointestinal (4%) and dyslipidemia (3%). Two hundred and fifty two (47%) reported no side effects and for the rest data were lacking. Only 165 (56%) of the latter were in the “Ongoing” group at the end of the study, as compared to an overall patient representation of 71% in that group. Less than half of the patients with side effects (28 of 60) stopped LPV/r because of those effects as reported by the clinician (Table 2), comprising only about 16% of all the 177 who discontinued treatment. The other known reasons for stopping LPV/r were death (7%, 13 patients); non-adherence (14%); drug resistance to LPV/r – 31 patients (17%) developed resistance to the drug but only 14 (8%) reportedly stopped solely because of this; end of pregnancy (6%, 10 women); several technical/logistical reasons (e.g., refrigeration problems); and loss to follow-up (11%, 19 patients). Nine patients (5%) refused to continue taking the drug, despite good clinical results, for none of the above reasons. Forty six percent of MSM stopped the treatment with LPV/r, significantly more than in the other groups (26%–30%; p = 0.008).

**Table 2:** Side effects and other reasons for stopping LPV/r treatment.

A	Main Reasons for stopping LPV/r treatment		
		No	%
	Side effects	28	16
	Non adherence	25	14
	Technical reasons and lost to follow up	19	12
	Death	13	7
	Resistance	14	8
	PMTCT	10	6
	Patient refusal	9	5
	Immunological failure	1	1
	Not reported	58	29
	Total number of patients stopping LPV/r treatment	177	100

B	Side effects		
		No	%
	Gastrointestinal	23	4
	Dislypidemia	17	3
	Lipodystrophy	9	1
	Dermatological	4	0.5
	CNS	1	0.2
	Others	5	1
	Total number of patients with side effects	60	10
	No Side effects	252	42
	ND regarding side effects – Ongoing treatment	165	27
	ND regarding side effects – Stopped treatment	130	21
	Total number of LPV/r treated patients	607	100

(A) Reasons for stopping LPV/r treatment as reported by physicians. Although samples from 31 patients were resistant to LPV/r, only for 14 it was the only reason for stopping the treatment. Technical reasons include refrigeration problems, inability to increase volume of syrup, travel, unavailability for follow-up, *etc.* (B). Side effects reported by physicians.

Abbreviations: CNS –Central Nervous System; ND – no data; PMTCT – treatment during pregnancy only, to Prevent Mother to Child Transmission.

### Viral load and CD4

Viral load and CD4 results are described in Tables 3 and 4. Patients were stratified according to subtype (B, C and Non-BC), gender (Female or Male), PI experience prior to LPV/r (first PI or second or higher), and to whether they were on LPV/r treatment (Ongoing group) or had stopped it during the study. As most subtype B patients were MSM, this patient population was also compared to subtype-C males (M_C). For the whole cohort median VL was 64,200±47,036 copies/ml (4.81 logs, range <25 to 19,000,000) at the initiation of treatment with LPV/r and 399±16,845 copies/ml (2.60 logs, range <25 to 9,000,000) at the end of the study. The median decrease in VL was 1.9±0.1 logs (range −1.8 to +5.1), and 74.1% of patients were below detection level at the end of the study. Median CD4 count was 186±8.9 (1 to 1,421) cells/µl at treatment initiation and 341±11.3 [2 to 2,193] cells/µl at the end, with a median increase of 121±8.4 [−6 to 1,693] cells/µl.

**Table 3:** Demographic and clinical data of patients classified into groups.

Group	Gender		Age (years)	F-UP (years)	Time on LPV/r (months)	Ongoing (%)	Stopped (%)	VL<LDL at the end (%)	*P values* a		
(No. of Patients)	F (%)	M (%)							Time on LPV/r	Ongoing	LDL
Total (607)	242 (40)	365 (60)	38.2±0.5	5.2±0.2	23.7±0.8	71	29	74.1			
B (139)	20 (14)	119 (86)	38.5±0.9	6.3±0.5	24.4±1.6	54	45	78.4	B *vs.* C or B *vs.* non-BC		
C (391)	199 (51)	192 (49)	38.1±0.7	5.6±0.3	23.1±1.0	76	24	71.9	0.8	0.001	0.1
Non-BC (77)	23 (30)	54 (70)	37.7±1.3	2.5±0.5	27.0±2.1	73	27	77.9	0.4	0.009	0.9
M (365)	0 (0)	365 (100)	39.4±0.7	5.3±0.3	24.1±1.0	69	31	72.9	M *vs.* F		
F (242)	242 (100)	0 (0)	35.6±0.9	5.1±0.4	22.9±1.2	74	26	76	0.5	0.2	0.6
Ongoing (429)	178 (41)	251 (59)	38.3±0.7	5.3±0.3	27.0±1.0	100	0	81.1	Ongoing *vs.* Stopped		
Stopped (177)	64 (36)	113 (64)	37.6±0.9	5.2±0.4	14.5±1.2	0	100	57.1	0.01	<0.0001	<0.0001
1^st^ PI (300)	123 (41)	177 (59)	37.5±0.7	2.2±0.3	21.9±0..9	68	32	81	1^st^ PI *vs.* 2^nd^ _PI		
2^nd^ _PI (307)	119 (39)	188 (61)	38.9±0.8	7.6±0.3	25.1±1.2	73	27	67.4	<0.0001	0.3	<0.0001
MSM (75)	0 (0)	75 (100)	37.8±1.1	3.8±0.5	25.0±2.0	56	44	80	MSM *vs.* C_M		
C_M (192)	0 (0)	192 (100)	40.0±1.1	6.2±0.3	23.8±1.4	76	24	69.1	0. 04	0.0001	0.06

Patients were grouped according to subtype (B, C and non-BC), to Gender (F or M), to whether LPV/r was first PI, second, or later-given PI, and to whether they were on treatment (Ongoing) at the end of the study or had stopped. As most subtype B patients were MSM, this group was also compared to subtype C males.

Abbreviations: 1st_PI – patients receiving LPV/r as first PI; 2nd _PI – patients receiving LPV/r as second or higher-order PI; B – patients with HIV subtype B; C – patients with HIV subtype C; C_M – male patients with subtype C; F –female; F-UP – follow up, time from diagnosis to start of the study, in years; M– male; MSM – men who have sex with Men; non-BC- patients with HIV subtype other than subtype B or C; PI – protease inhibitor; VL – viral load; <LDL - below detection level.

a *p* values were calculated between groups in the box.

The C or non-BCgroups were compared to the B group.

**Table 4:** Viral load and CD4 counts at the beginning and the end of the study.

	VL_start	VL_end	ΔVL					*P values* a		
Group	cp/ml ± SEM	cp/ml ± SEM	cp/ml ± SEM	Log_ΔVL	CD4_Start	CD4_Last	ΔCD4^b^			
(No. of Patients)	Log(cp/ml) ±SEM	Log(cp/ml) ±SEM	Log(cp/ml) ±SEM	Log (cp/ml) ±SEM	cells/µl ±SEM	cells/µl ±SEM	cells/µl ±SEM	ΔVL	*CD4 Start*	ΔCD4
	[Range]	[Range]	[Range]							
Total (607)	64,200±47,036	399±16,845	41,601±49,088							
	4.81±0.05	2.60±0.05	4.62±0.12	1.85±0.06	186±8.9	341±11.3	12±8.4			
	[25–19,000,000]	[25–9,000,000]	[−8.250,000±18,999,601]	[−1.79±5.06]						
	[1.40–7.28]	[1.40–6.95]	[−6.96±7.23]							
B (139)	42,000±39,941	199±66,632	36,601±73,6232	2.35±0.14	216±20.8	468±27.2	175±22.9		B *vs.* C	
	4.63±0.12	2.30±0.09	4.63±0.24					0.2	0.5	<0.0001
C (391)	55,700±63,584	399±11,001	37,975±63458	2.00±0.07	178±10.8	306±12.7	98±8.6	C *vs.* non-BC		
	4.76±0.06	2.60±0.05	5.00±0.16					0.2	0.8	<0.0001
Non-BC (77)	95,000±161,780	399±12,835	86,383±162,232	2.60±0.12	196±21.8	386±31.7	203±26.5	B *vs.* non-BC		
	4.98±0.15	2.60±0.12	5.00±0.29					0.1	0.7	0.5
M (365	70,000±50,705	399±27,787	49,240±55,734	2.08±0.08	165±11.5	313±14.1	122±10.0			
	4.85±0.07	2.60±0.06	4.70±0.16					M *vs.* F		
F (242)	45,300±89,546	399±6,377	32,815±89,426	1.60±0.09	220±13.9	372±18.4	121±14.7	0.6	0.02	0.5
	4.65±0.08	2.60±0.07	5.00±0.19							
Ongoing (429)	58,300±63,594	399±7,930	43,801±63,190	2.60±0.05	196±11.5	362±13.6	154±10.5			
	4.77±0.06	2.60±0.05	4.76±0.06					1^st^ PI *vs.* 2^nd^ _PI		
Stopped (177)	67400±45,934	399±54,874	36,700±67,961	1.54±0.13	176±12.4	250±19.0	61±12.8	<0.0001	<0.0001	0.005
	4.83±0.10	2.60±0.11	4.57±0.26							
1st PI (300)	80,400±79,259	399±10,389	74,801±74,453	2.27±0.09	175±10.1	340±14.4	162±11.0			
	4.91±0.08	2.60±0.06	4.90±0.16					1^st^ PI *vs.* 2^nd^ _PI		
2nd _PI (307)	47,300±56,585	399±31,807	27,651±63,581	1.48±0.08	196±14.3	346±17.3	92±12.6	<0.0001	<0.0001	0.005
	4.67±0.07	2.60±0.07	4.44±0.18							
MSM (75)	120,000±54,809	140±120,534	87,975±126,564	2.60±0.20	178±25.9	388±34.5	186±25.5			
	5.08±0.16	2.15±0.14	4.94±0.35					MSM *vs.* C_M		
C_M (192)	71,700±69,286	399±21,087	56,251±68,457	1.92±0.11	145±16.0	269±18.2	84±10.3	0.1	0.003	0.0008
	4.86±0.09	2.60±0.08	4.72±0.24							

Patients were grouped as in Table 3. The median of the individual CD4 changes, ΔCD4, was calculated for each group (rather than the difference between the medians of CD4 counts). Selected *p*-values between relevant groups are shown. Significant differences between groups in other parameters were not found.

Abbreviations: 1st_PI – patients receiving LPV/r as first PI; 2nd _PI – patients receiving LPV/r as second or higher-order PI; B – patients with HIV subtype B; C – patients with HIV subtype C; C_M – male patients with subtype C; F –female; F-UP – follow up, time from diagnosis to start of the study, in years; M– male; MSM – men who have sex with Men; non-BC- patients with HIV subtype other than subtype B or C; PI – protease inhibitor; VL – viral load; <LDL - below detection level.

a *p* values were calculated between groups in the box. The C or non-BC groups were compared to the B group.

b the median values of the individual ΔCD4 values of patients.

After stratification in terms of different groups as described we found, in particular, that the fraction of patients ending up below detection level did not differ significantly between subtypes B and C, despite the fact that treatment was interrupted significantly more frequently by B-group patients than by C (P = 0.001), and treatment interruption was strongly associated with a poorer virologic outcome (p<0.001; Table 3). Treatment parameters other than treatment interruption were unlikely to confound the subtype-based comparison because they did not significantly differ for the two subtypes. Importantly, there were no significant differences in the fraction of those receiving LPV/r as first PI or in LPV/r treatment duration (Table 1). Also, no statistical differences were found between the subtypes in the median levels at the starting and ending points or in the log decrease of viral load, as well as in the median CD4 count at starting and ending (Table 4). Yet, when we calculated the median of the individual increases in absolute CD4 counts, we found that it was significantly larger for B patients compared to C, despite the strong association of the rate of treatment interruption with ΔCD4 (p<0.0001; Table 4). This discrepancy was found also between C and non-BC patients (p<0.0001), but not between B and non-BC (p = 0.5; Table 4). Consistent with this finding, the increase in CD4 counts among patients who did not stop treatment and who ended up below detection level averaged 238 cells/µl in the B-subtype group, 262 in the non-BC and only 179 cells/µl in the C group (*p* = 0.08 and 0.03, respectively). Additional ΔCD4 differences seen between MSM and C-infected males and between males and females reflect the predominance of MSM among B-patients.

Overall, the best outcome of LPV/r was observed when it was first PI (median VL decrease 2.3 logs, in 81% of patients VL below detection level, and CD4 increase of 162 cells/µl). We also stratified patients by baseline VL and CD4 counts and compared clinical outcomes of the different groups. Patients were divided into three groups according to VL, and each VL group was subdivided according to the CD4 counts, creating altogether nine groups. The VL ranges were >100,000, 10,000–100,000 and <10,000 cp/ml, and for CD4 counts <200, 200–500 and >500 cells/µl (Table 5). No significant differences in the outcome were observed between the groups. In most cases, the viral load dropped below detection level and there was a similar increase in CD4 independently of initial viremia and CD4 depletion levels. The patients in Group 9, with VL initially already below detection level and median CD4 count >700 cells/µl (Table 5), remained within these ranges.

**Table 5:** Clinical outcome of LPV/r treatment of patients stratified according to different VL and CD4 levels at baseline.

VL (cp/ml)	>100,000			10,000–100,000			<10,000			*P*		
CD4 (Cells/µl)	<200	200–500	>500	<200	200–500	>500	<200	200–500	>500	Gr 1 *vs.* Gr 4	Gr 1 *vs.* Gr 7	Gr 1 *vs.* Gr 9
Group (Number)	Gr 1 (181)	Gr 2 (57)	Gr 3 (13)	Gr 4 (78)	Gr 5 (68)	Gr 6 (7)	Gr 7 (69)	Gr 8 (92)	Gr 9 (42)			
Time since diagnosis (Years; median)	5.2	3.4	3.7	5.7	5.6	8.8	4.4	5.7	6.9	0.4	0.3	0.2
Time on Kaletra (Months; median)	26.1	24.4	33.7	25.7	26.6	44.1	20.4	16.6	10.7	0.2	0.2	**0.03**
LPV 1st PI (%)	98(55)	32(55)	7 (58)	38(49)	27(40)	0	37(61)	45(51)	8(19)	0.4	0.4	**<0.0001**
VL_start (cp/ml; median)	513,000	282,500	390,000	44,700	38,450	47,300	2,140	518	399	**0.003**	**<0.0001**	**<0.0001**
VL_end (cp/ml; median)	399	399	225	399	399	399	399	399	399	0.6	0.8	1
VL dec (cp/ml; median)	463,101	270,891	389,508	37,501	31,251	35,201	1,703	119	0	0.3	0.2	0.6
CD4_ start (cells/µl; median)	77	284	649	108	276	542	121	298	718	0.2	0.5	**<0.0001**
Last CD4 (cells/µl; median)	263	490	949	215	425	852	231	423	710	0.5	0.6	0.1
ΔCD4 (cells/µl; median)	165	199	313	129	122	25	112	79	−6	0.6	0.5	**<0.0001**
Stopped (%)	49 (28)	19 (33)	6 (50)	27(35)	17 (25)	0	21 (34)	25 (29)	3 (7)	0.2	0.5	**<0.05**
Genotyped (% of total)	46(26)	15(26)	3(25)	24(31)	15(22)	2(29)	20(33)	6(7)	4(10)	0.4	0.3	**<0.0001**
Resistance to LPV/r (% of genotyped)	13(28)	3(20)	1(33)	9(38)	2(4)	1(50)	2(20)	3(50)	1(25)	0.4	0.5	1
Side Effects	13(7)	4(7)	0	7(9%)	4(6)	0	7(12.5)	13(15.5)	5(12)	0.6	0.3	0.4
Exitus	8 (4)	0	0	2(3%)	2(3)	0	0	1(1)	0	0.7	0.2	0.4

Patients were stratified according to VL: above 100,000 cp/ml; between 10 and 100 thousands cp/ml and below 10,000 cp/ml when starting LPV/r. Each group was further divided according to baseline CD4 count: below 200 cells/µl; between 200 and 500 cells/µl and above 500 cell/µl, creating altogether 9 groups. Group 9 (VL below 10,000 cp/ml and CD4 counts more than 500 cell/µl) was significantly different from the other groups in two parameters: shorter time on LPV/r treatment and lower percent of patients receiving LPV/r as first PI. As the median VL of this group was initially below detection level and baseline CD4 count above 700, no further decline in viral load could be observed and the lack of further increase in CD4 was also expected.

cp/ml – copies/ml; Gr – group; VL –Viral Load.

### Resistance to LPV/r

Three hundred samples from 237 patients on LPV/r with VL ≥1,000 copies/ml were genotyped. The latest sample from each patient while on LPV/r was used in the analysis. Mutations and common polymorphisms are depicted in Table 6. The most common resistance mutation for all subtypes was I54V (in 70% of samples with mutations) followed by V82A (61%) and M46I (47%). The frequencies of I13V, I62V, L63P/A, V77I and I84V were significantly higher in B than in C and those of I15V, L19I, K20R, M36I, R41K, H69Q/D/R, T74S, and I93L higher in C as compared to B (p<0.01 to p<0.0001). After Bonferroni correction for multiple comparisons the difference in the prevalence of V82A/C between subtypes was insignificant. In addition, significantly higher frequencies of modifications I15V, L19I, and R41K were found in C (p = 0.03 to <0.0001; Table 6). Notably, in 114 (48%) of those failing LPV/r containing regimens, no resistance mutations were detected including mutations of the NRTI backbone (Table 7).

**Table 6:** Mutations and polymorphisms in samples genotyped under LPV/r treatment.

		Samples failing LPV/r as first PI n = 114						Samples failing LPV/r as second or higher PI n = 126						*p*								
		B n = 22		C n = 67		nonBC n = 25		B n = 21		C n = 89		non-BC n = 13		LPV/r first PI			LPV/r second or higher PI			First *vs.* second or higher		
Samples revealing major PI mutations		0		7 (10%)		1 (4%)		10 (47.6%)		40 (44.9%)		8 (61.5%)		B *vs. C*	B *vs.* non-BC	C *vs.* non-BC	B *vs. C*	B *vs.* non-BC	C vs. non-BC	B	C	non-BC
PI Mutations		No	%	No	%	No	%	No	%	No	%	No	%									
**I**	**L10F/I/V**	2	9	8	12	6	24	11	52	36	40	6	46							0.003	<0.00001	
	G16E	0	0	9	13	2	8	0	0	8	9	0	0	0.1								
	**K20R/I/M/T**	3	14	15	22	9	36	3	14	38	43	7	54		0.1		0.02	0.02			0.01	
	**L24I**	0	0	0	0	0	0	1	5	3	3	3	23						0.03			0.03
	***L33F***	0	0	0	0	0	0	2	10	8	9	2	15								0.01	
	M36I	4	18	64	96	22	88	8	38	88	99	10	77	<0.00001	<0.00001		<0.00001	0.04	0.006			
	K43T	0	0	0	0	0	0	0	0	1	1	0	0									
	***M46I/L***	0	0	4	6	1	4	6	29	23	26	3	23							0.009	0.001	0.1
	I47A/V	0	0	0	0	0	0	0	0	2	2	0	0									
	G48V/M	0	0	0	0	0	0	1	5	1	1	2	15						0.04			
	I50V/L	0	0	0	0	0	0	0	0	2	2	1	8									
	***F53L***	0	0	0	0	0	0	1	5	1	1	2	15						0.04			
	**I54A/V**	0	0	4	6	1	4	6	29	28	31	6	46							0.009	<0.00001	0.004
	Q58E	0	0	0	0	0	0	0	0	3	3	1	8									
	***L63P***	12	55	23	34	11	44	16	76	37	42	10	77				0.007	0.01	0.03	0.1		0.09
	***A71V***	3	14	2	3	1	4	6	29	15	17	3	23	0.09							0.008	0.1
	G73A/C/S/T	0	0	1	1	0	0	2	10	2	2	1	8									
	T74P/S	0	0	7	10	1	4	0	0	26	29	0	0		0.002		0.003		0.04		0.005	
	L76V	0	0	1	1	0	0	1	5	7	8	2	15								0.1	0.1
	**V82A/C/S**	0	0	6	9	1	4	3	14	26	29	6	46		0.002			0.06		0.1	0.002	0.004
	**I84V**	0	0	0	0	0	0	4	19	4	4	2	15	0.04			0.07			<0.05	0.1	0.1
	L89V	0	0	1	1	0	0	0	0	3	3	0	0									
	***L90M***	0	0	1	1	0	0	6	29	18	20	1	8							0.009	<0.00001	
**II**	V11I	0	0	1	1	0	0	1	5	1	1	0	0									
	I13V	4	18	2	3	11	44	8	38	10	11	3	23	0.03	0.07	<0.00001	0.006				0.07	
	L23I	0	0	0	0	0	0	0	0	2	2	1	8									
	D30N	0	0	0	0	0	0	0	0	1	1	0	0									
	D60E	0	0	5	7	3	12	3	14	3	3	4	31				0.08		0.005	0.1		
	I62V	14	64	5	7	4	16	9	43	13	15	4	31	<0.00001	0.001		0.01					
	H69K/Q/R/N	2	9	67	100	20	80	5	24	89	100	7	54	<0.00001	<0.00001	0.001	<0.00001		<0.00001			0.1
	V77I	7	32	2	3	1	4	7	33	1	1	3	23	0.001	0.02		<0.00001		0.006			0.1
	N83D	0	0	0	0	0	0	0	0	1	1	0	0									
	I85V	0	0	1	1	0	0	0	0	5	6	0	0									
	N88D/S	0	0	0	0	0	0	2	10	4	4	0	0								0.1	
	I93L	6	27	67	100	12	48	10	48	87	98	5	38	<0.00001	<0.00001	<0.00001	<0.00001		<0.00001			
**III**	T12S	0	0	13	19	1	4	2	10	22	25	0	0	0.03	0.01				0.08			
	I15V	2	9	29	43	3	12	2	10	57	64	4	31	0.004	<0.00001	0.006	<0.00001		0.03		0.01	
	L19I/K/M/T/V	1	5	18	27	2	8	3	14	30	34	3	23	0.03	0.01	0.09	0.1					
	E35D	10	45	6	9	15	60	4	19	16	18	6	46	<0.0001	0.01	<0.0001			0.03	0.1		
	S37N	4	18	29	43	9	36	6	29	51	57	8	62	0.04			0.03	0.08			0.1	
	R41K	3	14	64	96	21	84	4	19	85	96	8	62	<0.00001	<0.00001	0.08	<0.00001	0.03	0.001			

Samples from 237 patients under LPV/r treatment were analyzed. The last available sample from each patient was used. Mutations were divided into three groups according to: I - amino-acids that appear in at least one algorithm for LPV/r resistance (reviewed in [39]); II - other amino-acids known to be involved in resistance to PI; III - amino-acids that are not known to be involved in resistance to PI for which a statistically significant difference between subtypes was found. Amino acids that appear in all four algorithms are shown in bold type and those that appear in 3 different algorithms in italics. For mutation that are significantly different between subtypes the most prevalent variant is shown in bold type. In order to emphasize significant differences only *p* values smaller than 0.1 were noted. Mutations V32I, E34Q (Group I) and E35G (Group II) did not appear at all and were taken out from the table.

B – subtype B; C – subtype C; LPV/r – Lopinavir/ritonavir; non-BC– subtypes other than B or C; PI – protease Inhibitor.

**Table 7:** Resistance status of patients failing LPV/r.

	B	C	non-BC	Total	*p*		
	n = 43 (18%)	n = 156 (66%)	n = 38 (16%)	n = 237 (%)	B *vs.C*	B *vs.*non-BC	C *vs.*non-BC
Sensitive to all drugs (%)	28 (65)	65 (42)	21 (55)	114 (48)	0.009	0.5	0.1
LPV/r sensitive but resistant to backbone (%)	5 (12)	46 (29)	8 (21)	59 (28)	0.02	0.4	0.4
LPV/r sensitive (%)	34 (79)	111 (71)	29 (76)	174 (73)	0.3	0.4	0.7
LPV/r partially resistant (%)	2 (4)	8 (5)	1 (4)	11 (5)	1	0.9	0.9
LPV/r resistant (%)	7 (16)	37 (24)	8 (21)	52 (22)	0.4	0.8	0.8
Total	43	156	38	237			

Clinical resistance status of 237 patients failing cART including LPV/r was determined according to the True-gene™ software (version 10). The last available sample under LPV/r from each patient was used.

We evaluated the clinical significance of the resistance sequences using the Stanford University HIV Drug Resistance Database. Out of the 237 patients tested, only 52 (22%) were fully resistant to LPV/r, 11 (5%) were partially resistant and 174 (73%) were sensitive, half of those to all available PIs. Of the 63 samples with resistance to LPV/r, 1 (2%), 23 (36%) and 10 (16%) samples, respectively, were fully sensitive to the newer PIs atazanavir/r, darunavir/r or tipranavir/r. The major factor influencing accumulation of mutations was the history of PI usage. When LPV/r was the first PI administrated resistance was found in 8.7% of samples, similar to Barber *et al.* [48] and van Zyl *et al. (p = 0.5)* [33]. On the other hand, when LPV/r was the second or later PI in the sequence of treatment regimens 46% of the samples showed drug resistance. No difference between B and C patients was found in the percentage of LPV/r-resistant samples (*p* = 0.1–0.7) although significantly more subtype B samples were sensitive to all drugs (p = 0.009; Table 7).

## Discussion

LPV/r was incorporated into cART regimens in Israel in 2001. Here we retrospectively evaluated our experience with this drug gained over the following several years, involving 607 patients treated with LPV/r for an average of two years (range 3 to 95 months). We compared the rates of achieving suppression of viral replication (VL<400 copies/ml) and median CD4 increases, as well as drug resistance pathways, in those infected with different HIV-1 subtypes, mainly C and B.

We did not find significant differences between the major subtypes, B and C, in the rate of viral suppression, but there were such differences in the median increase in CD4-cell count, in particular in those who achieved undetectable viral load (Tables 3 & 4). Several factors could have contributed to these differences in the treatment efficacy: structural differences between the subtypes affecting their interaction with the drugs; different levels of adherence; different time on LPV/r treatment; different treatment history; differences in baseline VL and/or CD4-counts values, or genetic differences between the populations. Similar results were found by De Wit *et al.* comparing CD4 recovery of subtype A- and subtype B-infected patients [57]. It is of note that, also in that study, almost all of the non-B infected persons were heterosexuals of African origin while most B patients were MSM and Caucasians. It is beyond the scope of the present study to further investigate this interesting point. As revealed by stratification of the total population into subgroups, LPV/r treatment interruption and previous PI-treatment experience were strongly correlated to worse virologic and clinical results. But subtype-groups did not differ significantly in terms of previous PI experience. Median treatment time and baseline values were also similar for the different subtypes.

The different subtypes appear to have selected somewhat different pathways to replicate in the presence of the drug (Table 6). Samples from 237 patients who had viral load ≥1,000 copies/ml while on LPV/r were sequenced and analyzed. B patients had significantly higher prevalence, in comparison to non-B, of mutations I84V (*p = 0.02*), L63P and A71V (*p* = 0.04–0.001). T74S/A appeared in 28% of C samples and none of B (p<0.001). Almost all subtype-C patients had M36I and L89M which are in the consensus wild-type sequence of subtype C [12]. A significantly higher prevalence of modifications that are not known to confer resistance were found in treated C-virus in positions I15V, L19I, and R41K and in treated A1-virus in position E35D (p = 0.03 to<0.001; Table 6). There was a striking difference in the accumulation of mutations when LPV/r was used as first PI compared to when it was used as a second or higher PI. Moreover, failing treatment without mutations was most frequently observed when the LPV/r was used as first PI (Table 6). Using the TrueGene™ algorithm, 65% of the virologically failing patients who were tested were found to be sensitive to all drugs including RT inhibitors, and additional 11% were resistant to NRTI and/or NNRTI but sensitive to LPV/r. Only 17% of virologically failing patients were fully resistant and 6% partially resistant to LPV/r. No statistically significant difference was found in the percentage of resistant samples between B and C patients (*p* = 0.2–1; Table 7). In-vitro pharmacokinetic and pharmacodynamic data [58]–[61] showed that antiviral activity falls quickly as drug concentration is reduced for drugs with sharp dose-response curves and short half-lives, such as boosted protease inhibitors, limiting the time during which resistance can be selected for, enabling failure via growth of virus susceptible to the drug when adherence is poor. These studies, however, and the possibility that mutations may occur outside the protease-encoding gene [62]–[64], cannot satisfactorily explain virologic failure in the apparent absence of any mutations [53], [58], [65]–[68], including those related to NRTIs and NNRTIs. This phenomenon remains a conundrum.

**Figure 1:**
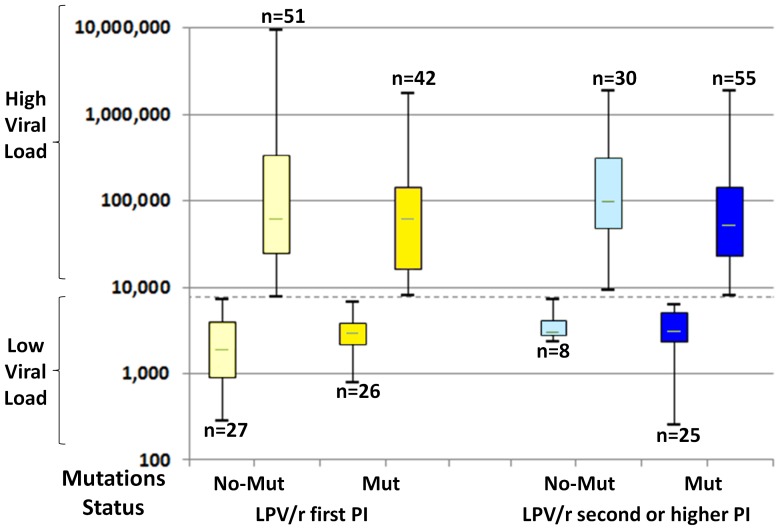
Partition of the viral-loads of treatment-failing patients into “high” and “low” categories. Shown are box-plots indicating partition of the viral-load distributions at treatment failure for those who failed either with or without mutations. Each plot shows median, quartiles and range. Viral load is expressed in copies/ml plasma. Dashed line – viral load cutoff, at 6,000 copies/ml. Mut – resistance conferring mutations; n – number; No-mut – No resistance conferring mutations; PI – Protease Inhibitor.

We stratified samples according to viral load and presence of resistance mutations. As reported briefly earlier, we found that both groups, those who failed with mutations and those who failed without them, could each be divided into two subgroups according to their viral-load levels (Fig. 1). Thus, there were patients failing with relatively low viral loads in the range of a few thousand copies/ml and others distinctively segregated within the hundred-thousand range. We had speculated [53] that in the higher range, a frequent cause of failure-without-mutations was that the patient's adherence was very poor indeed, while in the low VL range, though adherence may be far from optimal, drug concentrations were sufficient to partially suppress wild-type virus replication while the development of overt drug resistance under such conditions of partially suppressed replication could be delayed for weeks or months, due to existing genetic barriers and poor fitness of variants [69]. Alternatively, resistance in some cases might have existed but escaped detection, due to the limitation of the method of population sequencing, if several resistant species of the virus coexisted, each contributing below 15% of the total VL. The “high” and “low” dichotomy is found also in the VL of those failing with observable resistance mutations, but it had already been observed that maintaining the failing drug regimen often results in lower VL as compared to pretreatment levels [70].

Similar proportions of patients with different subtypes reported side effects (10.9%, 12.2% and 17.6% for B, C and non-BC, respectively; *p* = 0.4–0.8). It appears that the degree of success of the treatment in terms of viral load decrease and CD4 count increase as well as the actual presence or absence of side effects are not the only factors that influence the decision of patients to continue or discontinue treatment. For example, concerns regarding the risk of such side effects, especially in a more “educated” group, may be a factor that needs to be considered.

One earlier study [48] performed a comparison of a significant number (59) of genotyped subtype-C patients failing LPV/r-containing treatment with similar cohort of subtype-B patients at failure, where both groups were treated under equal conditions. No association of drug-resistance pattern with viral subtype was evident in that study. The larger number of genotyped subtype-C patients in our study (157) facilitated a more detailed comparison and the conclusion that the different subtypes select subtly different pathways to replicate in the presence of the drug (see above). Nevertheless, both studies support each other in showing an overall similarity in the patterns of resistance-conferring mutations. Importantly, we could estimate the total number of subtype-C infected persons in our cohort to a high precision, not just those who failed treatment, because of the almost perfect coincidence of subtype-C infection with an Ethiopian origin in the relevant period. Therefore, we could compare virological as well as immunological treatment outcomes for the two subtypes. Interpretation of the differences is confounded by the issue of adherence. In this regard, while the phenomenon of patients failing LPV/r-containing treatment in the apparent absence of resistance to the drug is well-recognized, we have documented also a surprisingly high frequency of failure with no evidence of resistance to the other simultaneously given drugs. Thus our study extends the work Barber *et al.* in terms of magnitude and scope.

With 157 C-subtype patients genotyped upon failure of LPV/r-including regimens, our study is now second only to van Zyl *et al.* in terms of information on drug-resistance mutations associated with such failure in subtype-C infected patients. These researchers studied trends in genotypic HIV-1 antiretroviral resistance in South Africa [33]. Their study included genotypic results from 486 subtype-C patients receiving cART who failed LPV/r given as first PI; LPV/r has now replaced previous PIs in cART regimens. At first sight, the fact that about half of the patients in our study received LPV/r as second PI may seem to diminish the relevance of our analysis of resistance mutations. However, although the frequency of virologic failures was higher in our study, there was a striking similarity between the two studies in the identity of the resistance-conferring mutations in treatment failing patients, in the relative overall frequencies in which they appeared, and in the frequencies of viruses harboring different numbers of mutations (between two and seven mutations). Moreover, as can be seen from Table 8, both studies suggest over-representation of variants possessing certain combinations of resistance mutations. While such associations did not reach statistical significance in each of the studies alone, pairwise-association analysis of all 101 C genotypes from LPV/r treatment-failing patients in the two studies together revealed a strong positive association trend between M46I and I54V (*p = 0.06*, Fisher's two-tailed Exact Test) and between I54V and V82A (*p = 0.09*). Consistent with these trends, the triplet M46I, I54V and V82A was very significantly over-represented (*p* = 0.007). On the other hand, L24I and L33F tend to appear mostly in conjunction with multiple other resistance mutations, as they do not confer strong resistance to LPV/r on their own but strengthen the effect of other mutations or the interaction between them once the latter have been selected.

**Table 8:** Mutation patterns in LPV/r failing C patients from Israel and South Africa.

Mut/Seq	Mutation Patterns	Number found	Origin (No.)				
1	M46I	2	SA(1), IL(1)	Most common patterns			
1	I47A/V	2	SA(1), IL(1)				
1	V82A	2	SA(1), IL(1)				
1	L90M	**7**	SA(1), IL(7)	Patterns	Actual frequency	Calculated frequency	*p*
2	L10F, V82A	5	SA(4), IL(1)				
2	M46I, L76V	2	SA(2)				
2	I54V, V82A	**7**	SA(6), IL(1)	M46I+L76V	0.19	0.12	0.2
3	L10F, I54V, V82A	2	IL(2)	M46I+V82A	0.47	0.37	0.2
3	M46I, I54V, V82A	6	SA(3), IL(3)	I54V+V82A	0.6	0.47	0.09
3	I54V, L76V, V82A	2	SA(2)	M46I+I54V+V82A	0.44	0.25	**0.01**
4	L10F, L33F, I54V, V82A	2	SA(1), IL(1)	M46I+I54V+L76V	0.17	0.08	0.09
4	L10F, M46I, I54V, V82A	4	SA(2), IL(2)	M46I+L76V+V82A	0.16	0.09	0.2
4	M46I, I50V, I54V, V82A	2	SA(2)	I54V+L76V+V82A	0.19	0.11	0.2
4	M46I, I54V, L76V, V82A	4	SA(3), IL(1)	M46I+I54V+L76V+V82A	0.16	0.06	**0.04**
5	L10F, M46I, I54V, L76V, V82A	7	SA(4), IL(3)				
6	L10F, L24I, L33F, M46I, I54V, V82A	3	SA(2), IL(1)				
Total	16	59					

The table lists all mutation patterns that appeared more than once in the combined dataset of 101 samples, 55 from South Africa (van Zyl *et al.* [33]) and 46 from Israel (this study). Fifty nine samples (58% of total) included 16 patterns. The actual frequency (number of times a pattern appeared in the dataset) and the calculated frequency (based on the overall frequencies of each mutation included in that pattern, assuming independence) for the most prevalent patterns are compared. *p*-values were calculated using Fisher's two- tailed Exact Test. IL – Israel; SA – South Africa.

### Study limitations

The retrospective nature of our study is a major limitation inherent to its design. We have attempted to provide subgroup analysis where possible. Another limitation is our inability to discern the effects of baseline structural differences between the subtypes on LPV/r impact from the effects of differences in adherence to, and persistence in, drug taking schedules prescribed by physicians. This difficulty exists because, although all patients are similarly treated by the same physicians, C patients largely belong to a group of immigrants from Ethiopia so that social and cultural factors may have affected adherence in ways that are hard to evaluate. Six out of seven AIDS treating centers in Israel participated in the study. Differences between the centers in keeping the patients on the drugs cannot be excluded, and since the ratio between B and C patients in each center varied, a bias could result. We believe, however, that such differences are secondary to the potential difference in adherence between subtype-B and subtype-C patients as groups. Assessing to what degree low-level residual viral replication contributed to differences in ΔCD4 was limited by the detection level of 400 copies/ml that we had to impose in the analysis as more sensitive tests were performed for only a fraction of the study population. Finally, as mentioned above, drug resistance might have escaped detection in some patients due to the limitations of the method of population sequencing. Ultra-deep sequencing would be required to assess this possibility.

In summary, our data highlight the long-term efficacy and safety of lopinavir/ritonavir among patients with both subtypes B and C. Despite the great variety in these populations in ethnic, educational and other socio-economic parameters, the differences in clinical outcome between the subtype groups appeared to have limited clinical relevance. These findings strongly support the expanding use of LPV/r in resource limited settings with high prevalence of subtype C infection.

## References

[1] UNAIDS (2013) UNAIDS Report on the Global AIDS Epidemic 2012. Available: http://wwwunaidsorg/en/media/unaids/contentassets/documents/epidemiology/2012/gr2012/20121120_UNAIDS_Global_Report_2012_enpdf.

[2] Hemelaar J, Gouws E, Ghys PD, Osmanov S, Isolation W-UNfH, et al (2011) Global trends in molecular epidemiology of HIV-1 during 2000–2007. AIDS 25: 679–689. 21297424 10.1097/QAD.0b013e328342ff93 PMC3755761

[3] Buonaguro FM, Buonaguro L, Del Gaudio E, Tornesello ML, Monaco M, et al (1996) V3 region genotyping of HIV isolates in northern Uganda: heteroduplex mobility assay, nucleotide sequence and phylogenetic analysis. Italian-Ugandan Cooperation AIDS Program. Antibiot Chemother 48: 39–48. 8726504

[4] Cornelissen M, van den Burg R, Zorgdrager F, Lukashov V, Goudsmit J (1997) pol gene diversity of five human immunodeficiency virus type 1 subtypes: evidence for naturally occurring mutations that contribute to drug resistance, limited recombination patterns, and common ancestry for subtypes B and D. J Virol 71: 6348–6358. 9261352 10.1128/jvi.71.9.6348-6358.1997 PMC191908

[5] Carr JK, Salminen MO, Albert J, Sanders-Buell E, Gotte D, et al (1998) Full genome sequences of human immunodeficiency virus type 1 subtypes G and A/G intersubtype recombinants. Virology 247: 22–31. 9683568 10.1006/viro.1998.9211

[6] Carr JK, Laukkanen T, Salminen MO, Albert J, Alaeus A, et al (1999) Characterization of subtype A HIV-1 from Africa by full genome sequencing. AIDS 13: 1819–1826. 10513639 10.1097/00002030-199910010-00003

[7] Quinnan GV Jr, Zhang PF, Fu DW, Dong M, Alter HJ (1999) Expression and characterization of HIV type 1 envelope protein associated with a broadly reactive neutralizing antibody response. AIDS Res Hum Retroviruses 15: 561–570. 10221533 10.1089/088922299311088

[8] Wu TD, Schiffer CA, Gonzales MJ, Taylor J, Kantor R, et al (2003) Mutation patterns and structural correlates in human immunodeficiency virus type 1 protease following different protease inhibitor treatments. J Virol 77: 4836–4847. 12663790 10.1128/JVI.77.8.4836-4847.2003 PMC152121

[9] Hirsch MS, Gunthard HF, Schapiro JM, Brun-Vezinet F, Clotet B, et al (2008) Antiretroviral drug resistance testing in adult HIV-1 infection: 2008 recommendations of an International AIDS Society-USA panel. Clin Infect Dis 47: 266–285. 18549313 10.1086/589297

[10] Rhee SY, Fessel WJ, Zolopa AR, Hurley L, Liu T, et al (2005) HIV-1 Protease and reverse-transcriptase mutations: correlations with antiretroviral therapy in subtype B isolates and implications for drug-resistance surveillance. J Infect Dis 192: 456–465. 15995959 10.1086/431601 PMC2597526

[11] Cane PA, de Ruiter A, Rice P, Wiselka M, Fox R, et al (2001) Resistance-associated mutations in the human immunodeficiency virus type 1 subtype c protease gene from treated and untreated patients in the United Kingdom. J Clin Microbiol 39: 2652–2654. 11427587 10.1128/JCM.39.7.2652-2654.2001 PMC88203

[12] Grossman Z, Vardinon N, Chemtob D, Alkan ML, Bentwich Z, et al (2001) Genotypic variation of HIV-1 reverse transcriptase and protease: comparative analysis of clade C and clade B. AIDS 15: 1453–1460. 11504976 10.1097/00002030-200108170-00001

[13] Averbuch D, Schapiro JM, Lanier ER, Gradstein S, Gottesman G, et al (2006) Diminished selection for thymidine-analog mutations associated with the presence of M184V in Ethiopian children infected with HIV subtype C receiving lamivudine-containing therapy. Pediatr Infect Dis J 25: 1049–1056. 17072129 10.1097/01.inf.0000243211.36690.d5

[14] Brenner B, Turner D, Oliveira M, Moisi D, Detorio M, et al (2003) A V106M mutation in HIV-1 clade C viruses exposed to efavirenz confers cross-resistance to non-nucleoside reverse transcriptase inhibitors. AIDS 17: F1–5. 12478089 10.1097/00002030-200301030-00001

[15] Ariyoshi K, Matsuda M, Miura H, Tateishi S, Yamada K, et al (2003) Patterns of point mutations associated with antiretroviral drug treatment failure in CRF01_AE (subtype E) infection differ from subtype B infection. J Acquir Immune Defic Syndr 33: 336–342. 12843744 10.1097/00126334-200307010-00007

[16] Grossman Z, Istomin V, Averbuch D, Lorber M, Risenberg K, et al (2004) Genetic variation at NNRTI resistance-associated positions in patients infected with HIV-1 subtype C. AIDS 18: 909–915. 15060438 10.1097/00002030-200404090-00008

[17] Grossman Z, Paxinos EE, Averbuch D, Maayan S, Parkin NT, et al (2004) Mutation D30N is not preferentially selected by human immunodeficiency virus type 1 subtype C in the development of resistance to nelfinavir. Antimicrob Agents Chemother 48: 2159–2165. 15155216 10.1128/AAC.48.6.2159-2165.2004 PMC415604

[18] Wainberg MA, Zaharatos GJ, Brenner BG (2011) Development of antiretroviral drug resistance. N Engl J Med 365: 637–646. 21848464 10.1056/NEJMra1004180

[19] World Health Organization (2013) Consolidated Guidelines on the use of Antiretroviral Drugs for Treating and Preventing HIV Infection. Available: http://wwwwhoint/hiv/pub/guidelines/arv2013/download/en/indexhtml: Accessed 2013 Jun 30.

[20] South African Government (2007) In: South African Guidelines, Factsheet Section 10, Antiretroviral. Government SA, editor South African Government 76–95.

[21] World Health Organization (2008) WHO Revised treatment recommendations for infants. WHO; Apr 10–11, 2008 pp. 1–10. 2008 ed.

[22] Falloon J, Piscitelli S, Vogel S, Sadler B, Mitsuya H, et al (2000) Combination therapy with amprenavir, abacavir, and efavirenz in human immunodeficiency virus (HIV)-infected patients failing a protease-inhibitor regimen: pharmacokinetic drug interactions and antiviral activity. Clin Infect Dis 30: 313–318. 10671334 10.1086/313667

[23] Ribaudo HJ, Haas DW, Tierney C, Kim RB, Wilkinson GR, et al (2006) Pharmacogenetics of plasma efavirenz exposure after treatment discontinuation: an Adult AIDS Clinical Trials Group Study. Clin Infect Dis 42: 401–407. 16392089 10.1086/499364

[24] King J, Aberg JA (2008) Clinical impact of patient population differences and genomic variation in efavirenz therapy. AIDS 22: 1709–1717. 18753940 10.1097/QAD.0b013e32830163ad

[25] Ribaudo HJ, Liu H, Schwab M, Schaeffeler E, Eichelbaum M, et al (2010) Effect of CYP2B6, ABCB1, and CYP3A5 polymorphisms on efavirenz pharmacokinetics and treatment response: an AIDS Clinical Trials Group study. J Infect Dis 202: 717–722. 20662624 10.1086/655470 PMC2919241

[26] Achan J, Kakuru A, Ikilezi G, Ruel T, Clark TD, et al (2012) Antiretroviral agents and prevention of malaria in HIV-infected Ugandan children. N Engl J Med 367: 2110–2118. 23190222 10.1056/NEJMoa1200501 PMC3664297

[27] Lockman S, Hughes M, Sawe F, Zheng Y, McIntyre J, et al (2012) Nevirapine- versus lopinavir/ritonavir-based initial therapy for HIV-1 infection among women in Africa: a randomized trial. PLoS Med 9: e1001236. 22719231 10.1371/journal.pmed.1001236 PMC3373629

[28] Taiwo B, Murphy RL, Katlama C (2010) Novel antiretroviral combinations in treatment-experienced patients with HIV infection: rationale and results. Drugs 70: 1629–1642. 20731472 10.2165/11538020-000000000-00000

[29] Hamers RL, Oyomopito R, Kityo C, Phanuphak P, Siwale M, et al (2012) Cohort profile: The PharmAccess African (PASER-M) and the TREAT Asia (TASER-M) monitoring studies to evaluate resistance–HIV drug resistance in sub-Saharan Africa and the Asia-Pacific. Int J Epidemiol 41: 43–54. 21071386 10.1093/ije/dyq192 PMC3304520

[30] Hamers RL, Sigaloff KC, Kityo C, Mugyenyi P, de Wit TF (2013) HIV-1 drug resistance in antiretroviral-naive patients in sub-Saharan Africa. Lancet Infect Dis 13: 196–197. 23427888 10.1016/S1473-3099(13)70012-4

[31] Levison JH, Orrell C, Gallien S, Kuritzkes DR, Fu N, et al (2012) Virologic failure of protease inhibitor-based second-line antiretroviral therapy without resistance in a large HIV treatment program in South Africa. PLoS One 7: e32144. 22427821 10.1371/journal.pone.0032144 PMC3302781

[32] Wallis CL, Mellors JW, Venter WD, Sanne I, Stevens W (2011) Protease Inhibitor Resistance Is Uncommon in HIV-1 Subtype C Infected Patients on Failing Second-Line Lopinavir/r-Containing Antiretroviral Therapy in South Africa. AIDS Res Treat 2011: 769627. 21490784 10.1155/2011/769627 PMC3066558

[33] Van Zyl GU, Liu TF, Claassen M, Engelbrecht S, de Oliveira T, et al (2013) Trends in Genotypic HIV-1 Antiretroviral Resistance between 2006 and 2012 in South African Patients Receiving First- and Second-Line Antiretroviral Treatment Regimens. PLoS One 8: e67188. 23840622 10.1371/journal.pone.0067188 PMC3694021

[34] ANRS AC11 Resistance Study Group (2012) HIV-1 genotypic drug resistance interpretation's algorithms, tables of rules 2012;. ANRS Version No. 22: ANRS AC11.

[35] Conradie F, Sanne I, Venter W, Eron J (2004) Failure of lopinavir-ritonavir (Kaletra)-containing regimen in an antiretroviral-naive patient. AIDS 18: 1084–1085. 15096819 10.1097/00002030-200404300-00024

[36] Kempf DJ, Isaacson JD, King MS, Brun SC, Xu Y, et al (2001) Identification of genotypic changes in human immunodeficiency virus protease that correlate with reduced susceptibility to the protease inhibitor lopinavir among viral isolates from protease inhibitor-experienced patients. J Virol 75: 7462–7469. 11462018 10.1128/JVI.75.16.7462-7469.2001 PMC114981

[37] Parkin NT, Chappey C, Petropoulos CJ (2003) Improving lopinavir genotype algorithm through phenotype correlations: novel mutation patterns and amprenavir cross-resistance. AIDS 17: 955–961. 12700444 10.1097/00002030-200305020-00003

[38] Friend J, Parkin N, Liegler T, Martin JN, Deeks SG (2004) Isolated lopinavir resistance after virological rebound of a ritonavir/lopinavir-based regimen. AIDS 18: 1965–1966. 15353986 10.1097/00002030-200409240-00016

[39] Maillard A, Chapplain JM, Tribut O, Bentue-Ferrer D, Tattevin P, et al (2007) The use of drug resistance algorithms and genotypic inhibitory quotient in prediction of lopinavir-ritonavir treatment response in human immunodeficiency virus type 1 protease inhibitor-experienced patients. J Clin Virol 38: 131–138. 17208042 10.1016/j.jcv.2006.11.011

[40] Diaz RS, Vasconcelos L, Hayden RL, Tenore S, Turcato G Jr, et al (2008) Similar efficacy of lopinavir/ritonavir-containing regimens among clades B and F HIV-1-Infected individuals in Brazil. J Acquir Immune Defic Syndr 47: 399–401. 18398975 10.1097/qai.0b013e31815b0d48

[41] Champenois K, Deuffic-Burban S, Cotte L, Andre P, Choisy P, et al (2008) Natural polymorphisms in HIV-1 protease: impact on effectiveness of a first-line lopinavir-containing antiretroviral therapy regimen. J Med Virol 80: 1871–1879. 18814256 10.1002/jmv.21315

[42] Lodwick RK, Smith CJ, Youle M, Lampe FC, Tyrer M, et al (2008) Stability of antiretroviral regimens in patients with viral suppression. AIDS 22: 1039–1046. 18520347 10.1097/QAD.0b013e3282fec415

[43] Murphy RL, da Silva BA, Hicks CB, Eron JJ, Gulick RM, et al (2008) Seven-year efficacy of a lopinavir/ritonavir-based regimen in antiretroviral-naive HIV-1-infected patients. HIV Clin Trials 9: 1–10. 18215977 10.1310/hct0901-1

[44] Pulido F, Delgado R, Perez-Valero I, Gonzalez-Garcia J, Miralles P, et al (2008) Long-term (4 years) efficacy of lopinavir/ritonavir monotherapy for maintenance of HIV suppression. J Antimicrob Chemother 61: 1359–1361. 18343802 10.1093/jac/dkn103

[45] Rhee SY, Taylor J, Fessel WJ, Kaufman D, Towner W, et al (2010) HIV-1 protease mutations and protease inhibitor cross-resistance. Antimicrob Agents Chemother 54: 4253–4261. 20660676 10.1128/AAC.00574-10 PMC2944562

[46] Lisovsky I, Schader SM, Martinez-Cajas JL, Oliveira M, Moisi D, et al (2010) HIV-1 protease codon 36 polymorphisms and differential development of resistance to nelfinavir, lopinavir, and atazanavir in different HIV-1 subtypes. Antimicrob Agents Chemother 54: 2878–2885. 20404123 10.1128/AAC.01828-09 PMC2897293

[47] Maroldo L, Fredrick LM, Robinson-Morgan K, Trinh R, Podsadecki TJ (2010) Efficacy and safety of lopinavir/ritonavir (LPV/r) in antiretroviral-experienced subjects infected with different subtypes of HIV-1. Journal of the International AIDS Society (Suppl 4) P30.

[48] Barber TJ, Harrison L, Asboe D, Williams I, Kirk S, et al (2012) Frequency and patterns of protease gene resistance mutations in HIV-infected patients treated with lopinavir/ritonavir as their first protease inhibitor. J Antimicrob Chemother 67: 995–1000. 22258921 10.1093/jac/dkr569

[49] Maayan S, Shinar E, Aefani M, Soughayer M, Alkhoudary R, et al (1994) HIV-1 prevalence among Israeli and Palestinian blood donors. AIDS 8: 133–134. 8011228 10.1097/00002030-199401000-00024

[50] Pollack S, Ben-Porath E, Fuad B, Raz R, Etzioni A (1994) Epidemiological and serological studies in HIV-infected Ethiopian immigrants to Israel. Acta Paediatr Suppl 400: 19–21. 10.1111/j.1651-2227.1994.tb13327.x 7833553

[51] Chemtob D, Grossman Z (2004) Epidemiology of adult and adolescent HIV infection in Israel: a country of immigration. Int J STD AIDS 15: 691–696. 15479507 10.1177/095646240401501011

[52] Levy I, Mor Z, Anis E, Maayan S, Leshem E, et al (2011) Men who have sex with men, risk behavior, and HIV infection: integrative analysis of clinical, epidemiological, and laboratory databases. Clin Infect Dis 52: 1363–1370. 21596678 10.1093/cid/cir244

[53] Avidor B, Turner D, Mor Z, Chalom S, Riesenberg K, et al (2013) Transmission Patterns of HIV-Subtypes A/AE versus B: Inferring Risk-Behavior Trends and Treatment-Efficacy Limitations from Viral Genotypic Data Obtained Prior to and during Antiretroviral Therapy. PLoS One 8: e57789. 23469241 10.1371/journal.pone.0057789 PMC3585963

[54] Liu TF, Shafer RW (2006) Web resources for HIV type 1 genotypic-resistance test interpretation. Clin Infect Dis 42: 1608–1618. 16652319 10.1086/503914 PMC2547473

[55] Kantor R, Machekano R, Gonzales MJ, Dupnik K, Schapiro JM, et al (2001) Human Immunodeficiency Virus Reverse Transcriptase and Protease Sequence Database: an expanded data model integrating natural language text and sequence analysis programs. Nucleic Acids Res 29: 296–299. 11125118 10.1093/nar/29.1.296 PMC29795

[56] Alcantara LC, Cassol S, Libin P, Deforche K, Pybus OG, et al (2009) A standardized framework for accurate, high-throughput genotyping of recombinant and non-recombinant viral sequences. Nucleic Acids Res 37: W634–642. 19483099 10.1093/nar/gkp455 PMC2703899

[57] De Wit S, Boulme R, Poll B, Schmit JC, Clumeck N (2004) Viral load and CD4 cell response to protease inhibitor-containing regimens in subtype B versus non-B treatment-naive HIV-1 patients. AIDS 18: 2330–2331. 15577548 10.1097/00002030-200411190-00016

[58] Bangsberg DR, Moss AR, Deeks SG (2004) Paradoxes of adherence and drug resistance to HIV antiretroviral therapy. J Antimicrob Chemother 53: 696–699. 15044425 10.1093/jac/dkh162

[59] Bangsberg DR, Porco TC, Kagay C, Charlebois ED, Deeks SG, et al (2004) Modeling the HIV protease inhibitor adherence-resistance curve by use of empirically derived estimates. J Infect Dis 190: 162–165. 15195256 10.1086/420790

[60] Shen L, Peterson S, Sedaghat AR, McMahon MA, Callender M, et al (2008) Dose-response curve slope sets class-specific limits on inhibitory potential of anti-HIV drugs. Nat Med 14: 762–766. 18552857 10.1038/nm1777 PMC2743464

[61] Rosenbloom DI, Hill AL, Rabi SA, Siliciano RF, Nowak MA (2012) Antiretroviral dynamics determines HIV evolution and predicts therapy outcome. Nat Med 18: 1378–1386. 22941277 10.1038/nm.2892 PMC3490032

[62] Dam E, Quercia R, Glass B, Descamps D, Launay O, et al (2009) Gag mutations strongly contribute to HIV-1 resistance to protease inhibitors in highly drug-experienced patients besides compensating for fitness loss. PLoS Pathog 5: e1000345. 19300491 10.1371/journal.ppat.1000345 PMC2652074

[63] Parry CM, Kohli A, Boinett CJ, Towers GJ, McCormick AL, et al (2009) Gag determinants of fitness and drug susceptibility in protease inhibitor-resistant human immunodeficiency virus type 1. J Virol 83: 9094–9101. 19587031 10.1128/JVI.02356-08 PMC2738216

[64] Gupta RK, Kohli A, McCormick AL, Towers GJ, Pillay D, et al (2010) Full-length HIV-1 Gag determines protease inhibitor susceptibility within in vitro assays. AIDS 24: 1651–1655. 20597164 10.1097/qad.0b013e3283398216 PMC2923069

[65] Vercauteren J, Deforche K, Theys K, Debruyne M, Duque LM, et al (2008) The incidence of multidrug and full class resistance in HIV-1 infected patients is decreasing over time (2001–2006) in Portugal. Retrovirology 5: 12. 18241328 10.1186/1742-4690-5-12 PMC2265747

[66] Audelin AM, Lohse N, Obel N, Gerstoft J, Jorgensen LB (2009) The incidence rate of HIV type-1 drug resistance in patients on antiretroviral therapy: a nationwide population-based Danish cohort study 1999–2005. Antivir Ther 14: 995–1000. 19918103 10.3851/IMP1412

[67] Di Giambenedetto S, Zazzi M, Corsi P, Gonnelli A, Di Pietro M, et al (2009) Evolution and predictors of HIV type-1 drug resistance in patients failing combination antiretroviral therapy in Italy. Antivir Ther 14: 359–369. 19474470

[68] Bartmeyer B, Kuecherer C, Houareau C, Werning J, Keeren K, et al (2010) Prevalence of transmitted drug resistance and impact of transmitted resistance on treatment success in the German HIV-1 Seroconverter Cohort. PLoS One 5: e12718. 20949104 10.1371/journal.pone.0012718 PMC2951346

[69] Grossman Z, Polis M, Feinberg MB, Grossman Z, Levi I, et al (1999) Ongoing HIV dissemination during HAART. Nature Medicine 5: 1099–1104. 10.1038/13410 10502799

[70] Deeks SG, Barbour JD, Grant RM, Martin JN (2002) Duration and predictors of CD4 T-cell gains in patients who continue combination therapy despite detectable plasma viremia. AIDS 16: 201–207. 11807304 10.1097/00002030-200201250-00009

